# Draft genome sequence of chloride-tolerant *Leptospirillum ferriphilum* Sp-Cl from industrial bioleaching operations in northern Chile

**DOI:** 10.1186/s40793-016-0142-1

**Published:** 2016-02-27

**Authors:** Francisco Issotta, Pedro A. Galleguillos, Ana Moya-Beltrán, Carol S. Davis-Belmar, George Rautenbach, Paulo C. Covarrubias, Mauricio Acosta, Francisco J. Ossandon, Yasna Contador, David S. Holmes, Sabrina Marín-Eliantonio, Raquel Quatrini, Cecilia Demergasso

**Affiliations:** Fundación Ciencia & Vida, Santiago, Chile; Centro de Biotecnología “Profesor Alberto Ruiz”, Universidad Católica del Norte, Antofagasta, Chile; Centro de Investigación Científica y Tecnológica para la Minería, Antofagasta, Chile; BHP Billiton Chile, Santiago, Chile; Facultad de Ciencias Biologicas, Universidad Andres Bello, Santiago, Chile

**Keywords:** *Leptospirillum ferriphilum*, Acidophilic, Iron oxidizing, Thermotolerant, Chloride tolerant, Bioleaching, Secondary copper sulfides, Atacamite

## Abstract

*Leptospirillum ferriphilum* Sp-Cl is a Gram negative, thermotolerant, curved, rod-shaped bacterium, isolated from an industrial bioleaching operation in northern Chile, where chalcocite is the major copper mineral and copper hydroxychloride atacamite is present in variable proportions in the ore. This strain has unique features as compared to the other members of the species, namely resistance to elevated concentrations of chloride, sulfate and metals. Basic microbiological features and genomic properties of this biotechnologically relevant strain are described in this work. The 2,475,669 bp draft genome is arranged into 74 scaffolds of 74 contigs. A total of 48 RNA genes and 2,834 protein coding genes were predicted from its annotation; 55 % of these were assigned a putative function. Release of the genome sequence of this strain will provide further understanding of the mechanisms used by acidophilic bacteria to endure high osmotic stress and high chloride levels and of the role of chloride-tolerant iron-oxidizers in industrial bioleaching operations.

## Introduction

Extremely acidophilic leptospirilli exhibit considerable physiological and genetic variation [1] and have been classified into four species groups according to 16S rRNA phylogeny [2–4]. Group I is represented by *Leptospirillum ferrooxidans*, Group II by *L. ferriphilum* and Group III by “*L. ferrodiazotrophum”* [5, 6]*.* Recently, metagenomic evidence has supported the recognition of a new species ascribed to Group IV [7].

As all leptospirilli, Group II members are aerobic and obligatly chemolithotrophic, ferrous iron oxidizing bacteria. However, they differ from the other groups in their G + C molar ratios, the number of copies of *rrn* genes and the size of 16S-23S rRNA gene spacers, as well as in their capacity to grow at 45 °C [5].

*L. ferriphilum* has been shown to be the dominant microorganism in commercial biooxidation tanks in South Africa [5] and in PLS from heap bioleaching processes in Chile [8–10]. *L. ferriphilum* Sp-Cl is a key biological member in industrial biomining applications, becoming the most abundant or even the exclusive microorganism in certain stages of processes involving ferrous iron oxidation [11, 12]. Competitive growth of *L. ferriphilum* Sp-Cl has been explained by the elevated temperature, particular electrochemical conditions and certain metal concentrations that develop during mineral leaching. *Leptospirillum* group II spp. have also been documented to act as the dominant primary producers on floating biofilms obtained from the Richmond Mine at Iron Mountain in USA [13, 14].

The genomes of three isolates of *L. ferriphilum* are available: the draft genome of the type strain DSM 14647 obtained from an acid mine drainage in Peru [15], the complete genome of strain ML04 isolated from acidic water near a hot spring in China [16] and the complete genome of strain YSK [NCBI NZ_CP007243] isolated from an acid mine drainage in China. In addition, draft genomes for other three Group II members, ‘C75’ [13], ‘5-way CG’ [17, 18] and ‘*L. rubarum*’ [19] have been derived from metagenomic studies of acid mine drainages in the USA, together with several genomic variants emerging on short time evolutionary scales [13].

This work reports the microbiological and genomic properties of the first industrial isolate of *L. ferriphilum**.* Strain Sp-Cl (DSM 22399) was isolated from the leaching solutions draining from bioleaching heaps at the Spence mine located in the Atacama Desert (northern Chile), where chalcocite is the major copper mineral and copper hydroxychloride atacamite [Cu_2_Cl(OH)_3_] is present in variable proportions in the ore. The dissolution of atacamite is the main source of chloride in the PLS of the leaching process at Spence mine, which ranges between 1.5 and 12.5 g L^−1^. The isolation of this industrially important, chloride tolerant, iron oxidizing acidophile is highly significant for both basic and applied reasons, being a relevant model for chloride leaching studies.

## Organism information

### Classification and features

**Fig. 1:**
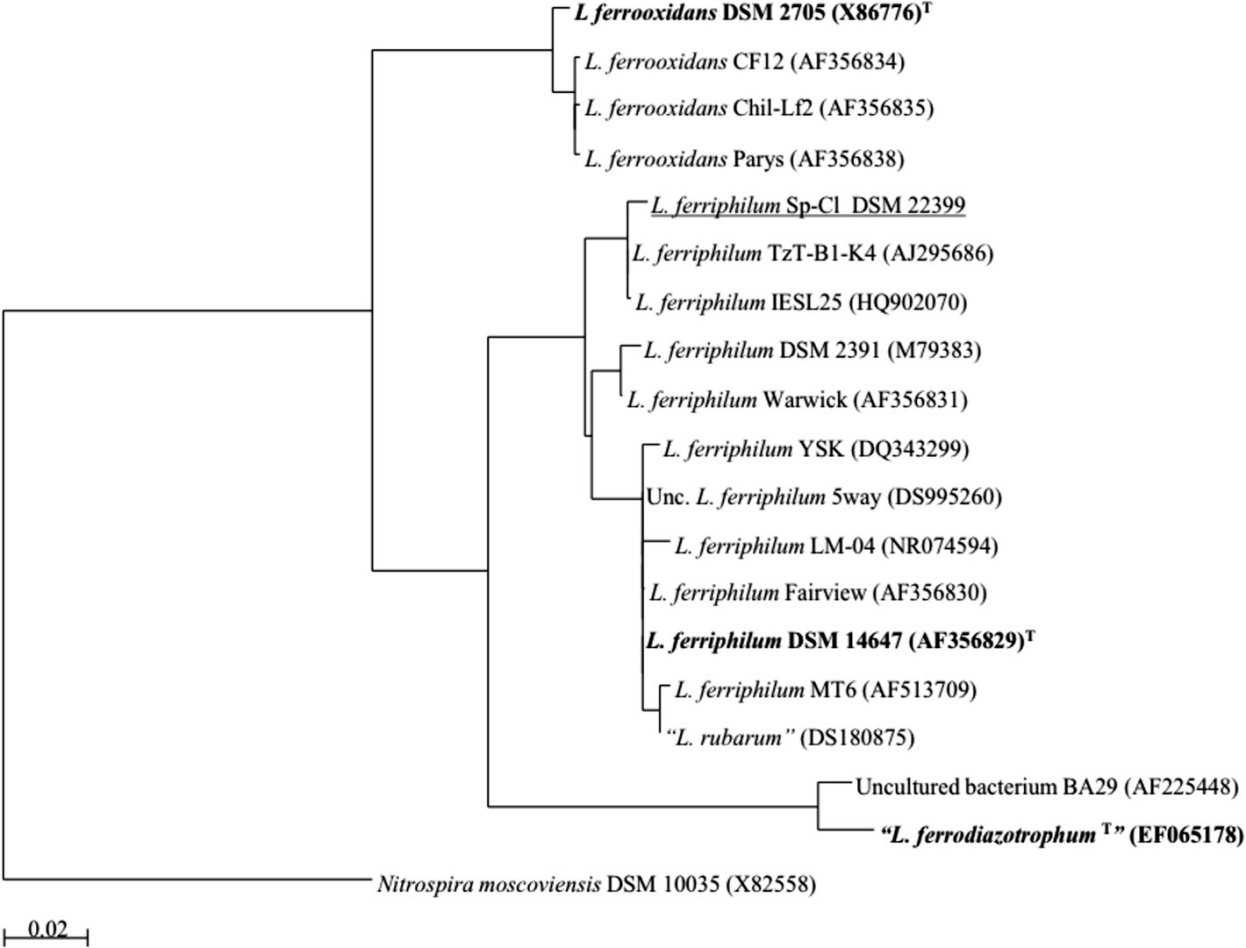
Phylogenetic affiliation of 16S rRNA gene sequences highlighting the position of *L. ferriphilum* strain Sp-Cl (underlined) relative to other type strains (bold) and non-type strains within the genus *Leptospirillum*. Database accession numbers are indicated between brackets (type strains = ^T^). The scale bar corresponds to 0.02 mutations per nucleotide position

**Fig. 2:**
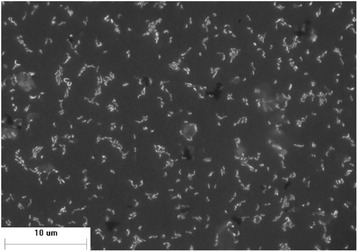
Confocal image of a culture of *L. ferriphilum* strain Sp-Cl stained with DAPI (4',6-diamidino-2-phenylindole)

Phylogenetic analysis of the 16S rRNA gene sequence of the isolate Sp-Cl, and other 17 isolates and/or clones representing currently recognized leptospirilli groups and species, revealed its close relation to *L. ferriphilum* (Fig. 1). *L. ferriphilum* Sp-Cl cells are morphologically very similar to other *L. ferriphilum* strains described previously [5, 15]. Sp-Cl cell are small sized (0.3 to 0.9 μm), curved rods (Fig. 2), depending on the culture state. The Gram stain for the Sp-Cl is consistently negative and a single polar flagellum enables its motility.

Like other known strains of the species, the Sp-Cl isolate utilizes ferrous iron as an energy source, but neither sulfur nor RISCs can be oxidized with energy conservation. It is also able to fix inorganic carbon (CO_2_) and nitrogen (N_2_) [20, 21]. The pH for growth ranges from 1.3 to 2.0 and the registered highest tolerated temperature is 45 °C, with an optimum between 30 and 37° (Table 1).

**Table 1:** Classification and general features of *Leptospirillum ferriphilum* Sp-Cl according to the MIGS recommendations [22]

MIGS ID	Property	Term	Evidence code^a^
	Classification	Domain *Bacteria*	TAS [38]
		Phylum “*Nitrospirae*”	TAS [38]
		Class “*Nitrospira*”	TAS [38]
		Order “*Nitrospirales*”	TAS [38]
		Family “*Nitrospiraceae*”	TAS [1, 38]
		Genus *Leptospirillum*	TAS [39]
		Species *Leptospirillum ferriphilum*	TAS [5]
		Strain Sp-Cl	TAS [5]
	Gram stain	Negative	TAS [5]
	Cell shape	Curved rod	IDA
	Motility	Motile	TAS [5]
	Sporulation	Non-spore forming	TAS [5]
	Temperature range	25° to 45 °C	NAS
	Optimum temperature	30° to 37 °C	NAS
	pH range, optimum	1.3 to 2.0; NA	IDA
	Carbon source	CO_2_	IDA
MIGS-6	Habitat	chloride, metal-rich and acidic environment	IDA
MIGS-6.3	Salinity	0-12 g/L Cl-	IDA
MIGS-22	Oxygen requirement	Aerobic	TAS [5]
MIGS-15	Biotic relationship	Free-living	IDA
MIGS-14	Pathogenicity	None	TAS [5, 22]
MIGS-4	Geographic location	Spence mine, Atacama Desert, Chile	IDA
MIGS-5	Sample collection	2007	IDA
MIGS-4.1	Latitude	22°.81 S	IDA
MIGS-4.2	Longitude	69°.26 W	IDA
MIGS-4.4	Altitude	1700	IDA

^a^Evidence codes - IDA: Inferred from Direct Assay; TAS: Traceable Author Statement (i.e., a direct report exists in the literature); NAS: Non-traceable Author Statement (i.e., not directly observed for the living, isolated sample, but based on a generally accepted property for the species, or anecdotal evidence). These evidence codes are from the Gene Ontology project [40]

Previous work on related *L. ferriphilum* strains has confirmed the greater tolerance to copper, silver and sulfate by this species as compared to *L. ferrooxidans* and ‘*L. ferrodiazotrophum’* members [10, 16, 22]. In addition, *L. ferriphilum* Sp-Cl has shown notable resistance to chloride (Cl^−^) and iron concentrations being able to oxidize ferrous iron (3 g/L) in the presence of Cl^−^ (12 g/L), making it a candidate for bioleaching with proportions of seawater [11, 12], which is an attractive opportunity in arid areas such as northern Chile and parts of Australia, or for chalcopyrite chloride leaching [23].

## Genome sequencing information

### Genome project history

The organism was selected for sequencing on the basis of its phylogenetic position and 16S rRNA similarity to members of the genus *Leptospirillum*. This Whole Genome Shotgun project has been deposited at GenBank under the accession LGSH00000000 [24]. The version described in this paper is the first version, LGSH01000000. Table 2 presents the project information and its association with MIGS version 2.0 compliance [25].

**Table 2:** Project information

MIGS ID	Property	Term
MIGS 31	Finishing quality	Draft
MIGS-28	Libraries used	GS FLX Titanium paired end libraries
MIGS 29	Sequencing platforms	Roche 454 GS FLX
MIGS 31.2	Fold coverage	20 ×
MIGS 30	Assemblers	Newbler 2.0.00.22
MIGS 32	Gene calling method	Glimmer 3.02
	Locus Tag	LGSH01000001-LGSH01000074
	Genbank ID	LGSH00000000
	GenBank Date of Release	31-12-2015
	GOLD ID	Gp0119878
	BIOPROJECT	PRJNA290892
MIGS 13	Source Material Identifier	PLS-Parcela-21
	Project relevance	Biomining, Tree of Life

### Growth conditions and genomic DNA preparation

*Leptospirillum ferriphilum* strain Sp-Cl (DSM 22399), was isolated from the PLS draining from a bioleaching heap at Spence mine, in the Antofagasta Region, Chile. The enrichment and isolation was performed at the Biotechnology Center (CBAR-UCN). Enrichment was performed using a PLS sample as inoculum followed by sequential dilutions and finally the culture was streaked on ABS solid media [26]. After repeated streaking of individual colonies growing on solid media an individual colony, designated Sp-Cl, was transferred to liquid medium.

The Sp-Cl strain was grown at 37 °C in liquid ABS medium (pH 1.5) containing 50 mM Fe2+ on an orbital shaker at 150 rpm. The DNA was isolated from cells collected on a nitrocellulose filter (0.22 μm pore), using a High Pure PCR Template Preparation kit according to the manufacturer’s instructions (Roche, Germany). The total amount of DNA was 10.4 μg (measured by Pico green assay). The quality of the DNA was assessed by agarose gel electrophoresis (0.8 % w/v).

### Genome sequencing and assembly

The genome of *L. ferriphilum* strain Sp-Cl was sequenced at Beckman Coulter Genomics using 454 sequencing technology and mate pair libraries with insert sizes of ~500 bp [27]. Pyrosequencing reads were assembled *de novo* using Newbler (v2.0.00.22). The final draft assembly contained 74 contigs in 74 scaffolds. The total size of the genome is ~2,5 Mbp and the final assembly is based on 61 Mbp of 454 data, which provides an average 20 × coverage of the genome.

### Genome annotation

Genes were identified using Glimmer 3.02 [28] as part of the RAST annotation pipeline [29]. The tRNA and tmRNA identification was achieved using ARAGORN v1.2.36 [30] and the rRNA prediction was carried out via HMMER3 [31]. Additional gene prediction analysis and functional annotation was performed at the Center for Bioinformatics and Genome Biology and at the Center for Biotechnology. The predicted CDSs were used to search the National Center for Biotechnology Information non-redundant database, UniProt, TIGRFam, Pfam, PRIAM, KEGG, COG and InterPro databases. Protein coding genes were analyzed for signal peptides using SignalP v4.1 [32] and transmembrane helices using TMHMM v2.0 [33].

## Genome properties

**Fig. 3:**
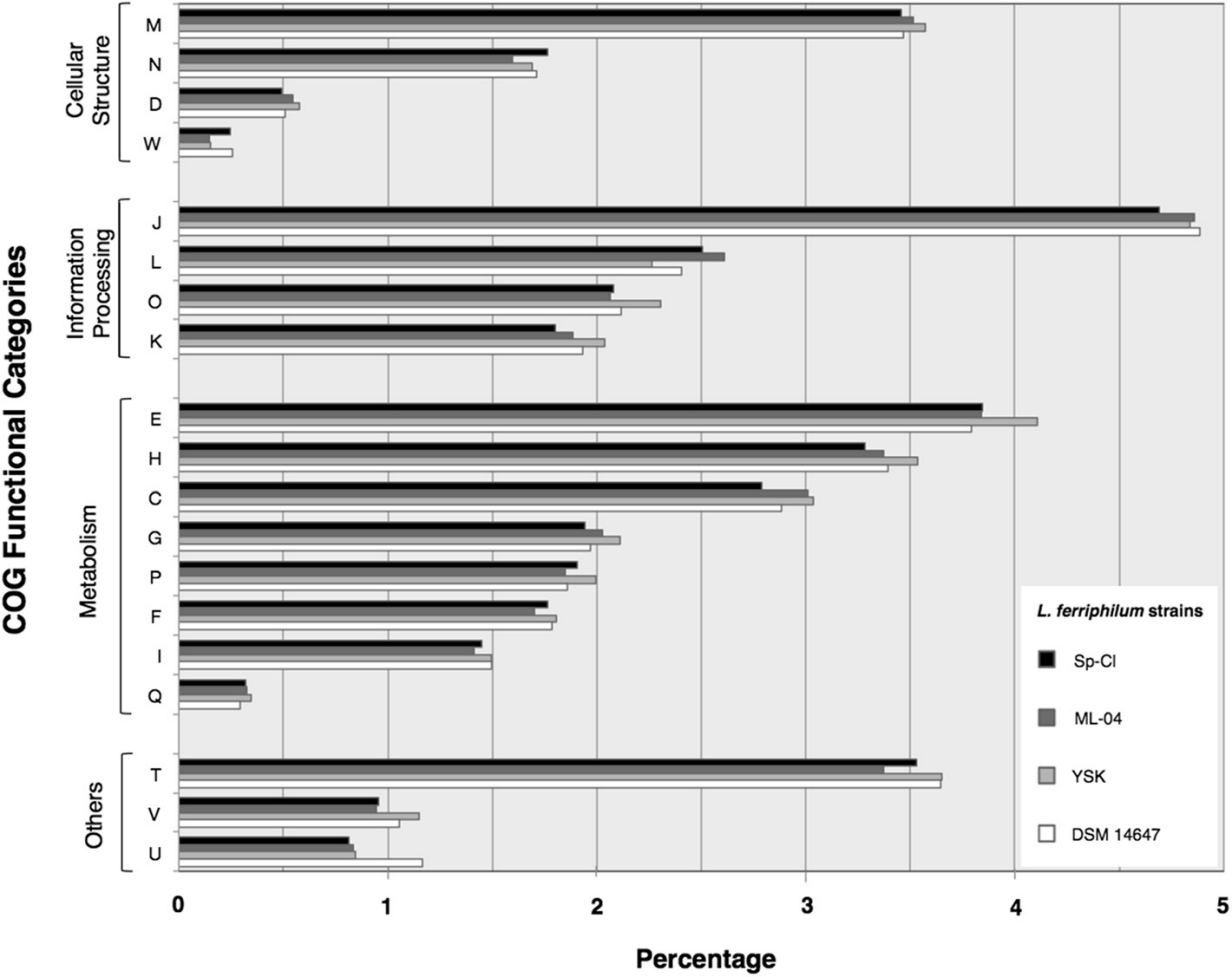
COG functional categories profiles in sequenced *L. ferriphilum* genomes. Values are expressed as percentages of the total protein complement of each strain. In black the Sp-Cl strain, in dark grey the ML-04 strain, in light grey the YSK strain and in white the type strain DSM 14647. COG categories codes for genes with assigned function are detailed in Table 4. W: Extracellular structures

The draft genome size is 2,475,669 nucleotides, with an average G + C content of 54.41 % (Table 3). From a total of 2,882 genes, 2,834 were protein coding genes and 48 are RNA genes. A total of 41.83 % of the genes were assigned a putative function while the remaining ones were annotated as hypotheticals. The distribution of genes into COGs functional categories for *L. ferriphilum* Sp-Cl is presented in Table 4 and its comparison against the other sequenced *L. ferriphilum* genomes is presented in Fig. 3.

**Table 3:** Genome statistics

Attribute	Value	% of Total^a^
Genome size (bp)	2,475,669	100.00
DNA coding (bp)	2,270,652	91.71
DNA G + C (bp)	1,347,012	54.41
DNA scaffolds	74	100.00
Total genes^b^	2,882	100.00
Protein coding genes	2,834	99.33
RNA genes^c^	48	1.66
Pseudo genes^d^	NA	NA
Genes in internal clusters	1,294	45.65
Genes with function prediction	1,631	56.59
Genes assigned to COGs	1,239	41.83
Genes with Pfam domains	1,778	61.69
Genes with signal peptides	221	7.66
Genes with transmembrane helices	633	21.96
CRISPR repeats	0	0.00

a) The total is based on either the size of the genome in base pairs or the total number of genes in theannotated genome.

b) Includes tRNA, tmRNA, rRNA.

c) Includes 23S, 16S and 5S rRNA.

d) n.d.: not determined

**Table 4:** Number of genes associated with general COG functional categories

Code	Value	% age^a^	Description
J	133	4.69	Translation, ribosomal structure and biogenesis
A	1	0.03	RNA processing and modification
K	51	1.79	Transcription
L	71	2.50	Replication, recombination and repair
B	1	0.03	Chromatin structure and dynamics
D	14	0.49	Cell cycle control, Cell division, chromosome partitioning
V	27	0.95	Defense mechanisms
T	100	3.53	Signal transduction mechanisms
M	98	3.45	Cell wall/membrane biogenesis
N	50	1.76	Cell motility
U	23	0.81	Intracellular trafficking and secretion
O	59	2.08	Posttranslational modification, protein turnover, chaperones
C	79	2.78	Energy production and conversion
G	55	1.94	Carbohydrate transport and metabolism
E	109	3.85	Amino acid transport and metabolism
F	50	1.74	Nucleotide transport and metabolism
H	93	3.28	Coenzyme transport and metabolism
I	41	1.44	Lipid transport and metabolism
P	54	1.91	Inorganic ion transport and metabolism
Q	9	0.31	Secondary metabolites biosynthesis, transport and catabolism
R	62	2.18	General function prediction only
S	36	1.27	Function unknown
-	1595	58.17	Not in COGs

^a^Percentages are based on the total number of protein coding genes in the genome

### Insights from the genome sequence

Genomic analysis of *L. ferriphilum* strains Sp-Cl allowed several genes involved in the three known trehalose biosynthetic pathways in bacteria to be identified (Table 5): GalU-OtsA-OtsB (I); TreY-TreZ-TreX (V) and TreS (IV) [34, 35]. Genes of IV and V synthetic pathways, considered as less-prominent routes for trehalose synthesis [36], were found in the genomes of *L. ferriphilum* DSM 14647^T^, and strains Sp-Cl and LF-ML04 in similar genomic contexts as well as in *A. ferrooxidans*. Similar organization has previously found in *Achromobacter xylosoxidans* and *Ralstonia eutropha* H16 (NCBI accession numbers NC_023061.1 and NC_008313.1, respectively), suggesting co-regulation between both pathways. The enzyme encoded by TreS can also produce maltose from either glycogen or malto-oligosaccharides and therefore TreS could also have glycogen debranching enzyme activity [36] and possibly maintain trehalose in equilibrium depending upon the osmotic requirement. In addition, another gene for a trehalose synthetase (Ble/Pep2) protein was located in the same genomic context in *L. ferriphilum* and strains Sp-Cl and LF-ML04 (Table 5) next to a gene for a maltosyltransferase (GlgE) in a similar configuration shown previously [34].

**Table 5:** Putative genes involved in threhalose synthetic pathway found in *L. ferriphilum* Sp-Cl genome (source NCBI)

Contig	Protein	Gene	Gene product
NZ_LGSH01000008	WP_038505518.1	*otsA*	Trehalose-6-phosphate synthetase (EC 2.4.1.15).
NZ_LGSH01000008	WP_038505520.1	*otsB*	Trehalose-6-phosphate phosphatase; anabolic (EC 3.1.3.12).
NZ_LGSH01000056	WP_053765286.1	*rpoS*	Putative two component, sigma54 specific, transcriptional regulator, Fis family.
NZ_LGSH01000044	WP_014959917.1	*galU-1*	Glucose-1 -phosphate-UDP-pyrophosphorylase (EC 2.7.7.9).
NZ_LGSH01000034	WP_014960519.1	*galU-2*	Glucose-1 -phosphate-UDP-pyrophosphorylase (EC 2.7.7.9).
NZ_LGSH01000035	WP_053764871.1	*treZ*	Malto-oligosyltrehalose trehalohydrolase (EC:3.2.1.141).
NZ_LGSH01000035	WP_053764870.1	*treY*	Malto-oligosyltrehalose synthase (EC 5.4.99.15).
NZ_LGSH01000049	WP_014962082.1	*treS*	Alpha amylase catalytic domain found in trehalose synthetase (EC 2.4.1.18).
NZ_LGSH01000035	WP_053764863.1	*ble/pep2-1*	Alpha amylase, probably involved in trehalose biosynthesis; Trehalose synthase (EC 5.4.99.16).
NZ_LGSH01000035	WP_014960479.1	*ble/pep2-2*	Alpha amylase, probably involved in trehalose biosynthesis; Trehalose synthase (EC 5.4.99.16).
NZ_LGSH01000035	WP_023525838.1	*ble/pep2-3*	Alpha amylase, probably involved in trehalose biosynthesis; Trehalose synthase (EC 5.4.99.16).
NZ_LGSH01000049	WP_014962082.1	*glgE*	Alpha amylase catalytic domain found in trehalose synthetase (EC 2.4.1.18).
NZ_LGSH01000055	WP_053765235.1	*treX/glgX-1*	Glycogen debranching enzyme (EC 3.2.1.-); 1,4-alpha-glucan-branching protein (EC 2.4.1.18).
NZ_LGSH01000009	WP_053764548.1	*treX/glgX-2*	Glycogen debranching enzyme (EC 3.2.1.-); 1,4-alpha-glucan-branching protein (EC 2.4.1.18).

Recently, genes for both trehalose and ectoine biosynthetic pathways were identified in the draft genome of the *L. ferriphilum* type strain DSM 14647 [15]. Transcriptomic studies of *L. ferrooxidans* strain L3.2 (isolated from the Rio Tinto, Spain) have pinpointed genes involved in the synthesis of trehalose, ectoine and systems for the transport of potassium in response to the increase of sulfate [37]. In addition, all of the components involved in trehalose and ectoine synthetic pathways have been identified in proteomic analysis performed in biofilms populated by *L. ferriphilum* and ‘*L. ferrodiazotrophum’* [19].

## Conclusions

The 2.4 Mbp draft genome sequence of *L. ferriphilum* strain Sp-Cl is arranged in 74 high quality scaffolds, resembling in size the type strain DSM 14647 and the Chinese strain ML-04. It encodes 2,834 protein-coding genes, 42 % of which were assigned putative functions, exceeding the predicted gene content of the type strain, the ML-04 strain and the YSK strain, and suggesting recent acquisition of additional functions. A total of 48 RNA genes partitioned into 44 tRNAs, 1 tmRNA and 1 rRNA operon. The most abundant COG functional category in *L. ferriphilum* strain Sp-Cl and all sequenced strains of the species were translation, ribosomal structure and biogenesis (J), amino acid and transport metabolism (E) and cell wall and cell membrane biogenesis (M). Release of the genome sequence of this strain will provide further understanding of the mechanisms used by acidophilic bacteria to endure high osmotic stress and high chloride levels and of the role of chloride-tolerant iron-oxidizers in industrial bioleaching operations.

## Abbreviations

PLS: pregnant leach solutions
RISCs: reduced inorganic sulfur compounds
